# Intractable Hiccups Caused by Diaphragmatic Eventration

**DOI:** 10.7759/cureus.24430

**Published:** 2022-04-24

**Authors:** Michelle K Hong, Albert Y Han, Jennifer L Long

**Affiliations:** 1 Head and Neck Surgery, University of California Los Angeles David Geffen School of Medicine, Los Angeles, USA; 2 Surgery and Perioperative Medicine, Veterans Administration Greater Los Angeles Healthcare System, Los Angeles, USA

**Keywords:** eventration, hiccups, voice therapy, diaphragmatic eventration, intractable hiccups

## Abstract

Intractable hiccups are a rare yet debilitating pathology with a broad differential and often indicate a more serious underlying pathology, which can range from neoplasms to structural abnormalities. In this case report, we present a 64-year-old male with seven months of intractable hiccups determined to be caused by eventration of the right hemidiaphragm. The patient was treated with baclofen to treat the hiccups pharmacologically. He was also prescribed voice therapy to establish rescue breathing techniques and reduce laryngospasm. Finally, he was referred to thoracic surgery for further evaluation and potential surgical intervention should his diaphragmatic eventration worsen or cause hypoxemia. To our knowledge, this is the first reported case of an association between diaphragmatic eventration and intractable hiccups. It is important to highlight this addition to the broad differential of intractable hiccups and to emphasize an interdisciplinary approach to workup and treatment of intractable hiccups.

## Introduction

A hiccup is an involuntary contraction of inspiratory muscles causing inspiratory airflow, which leads to a sudden reflexive closure of the glottis [1]. This sudden glottic closure is what causes the characteristic sound of a hiccup and is thought to be a protective reflex to prevent significant hyperventilation [1,2]. Hiccups are caused by gastric distension usually from ingestion of food or carbonated beverages [1,2]. They are most often acute in nature, a transient nuisance that self-resolves within a short time with no further complications. Intractable hiccups, on the other hand, are defined as hiccups lasting more than one month and are extremely distressing for patients [1,3-5]. These can be an indication of an underlying neoplasm or structural abnormality involving the diaphragm that requires further investigation. Our patient had an underlying musculoskeletal structural abnormality known as diaphragmatic eventration discovered on imaging. Here, we present the first known case of a patient whose intractable hiccups were caused by the eventration of the hemidiaphragm.

## Case presentation

A 64-year-old male veteran with a past medical history of hypertension, hyperlipidemia, and gastroesophageal reflux disease (GERD) presented to the otolaryngology clinic for further workup of his intractable hiccups. His hiccups began about seven months ago with no obvious inciting event. The hiccups are triggered by the use of voice and thus he has been using his voice less out of fear of embarrassing himself in public. Otherwise, there are no clear alleviating or exacerbating factors for the onset of his hiccup episodes. He denies dysphagia, odynophagia, or voice hoarseness.

He was initially seen by his primary care provider for his hiccups, who then referred him to gastroenterologists (GI), neurology, and psychiatry specialists with no resolution of his symptoms. GI performed an upper endoscopy, which did not show any abnormalities. He was prescribed anti-reflux therapy to treat his GERD, but his hiccups persisted. He was additionally trialed on baclofen with no symptomatic improvement before being referred to otolaryngology.

**Figure 1:**
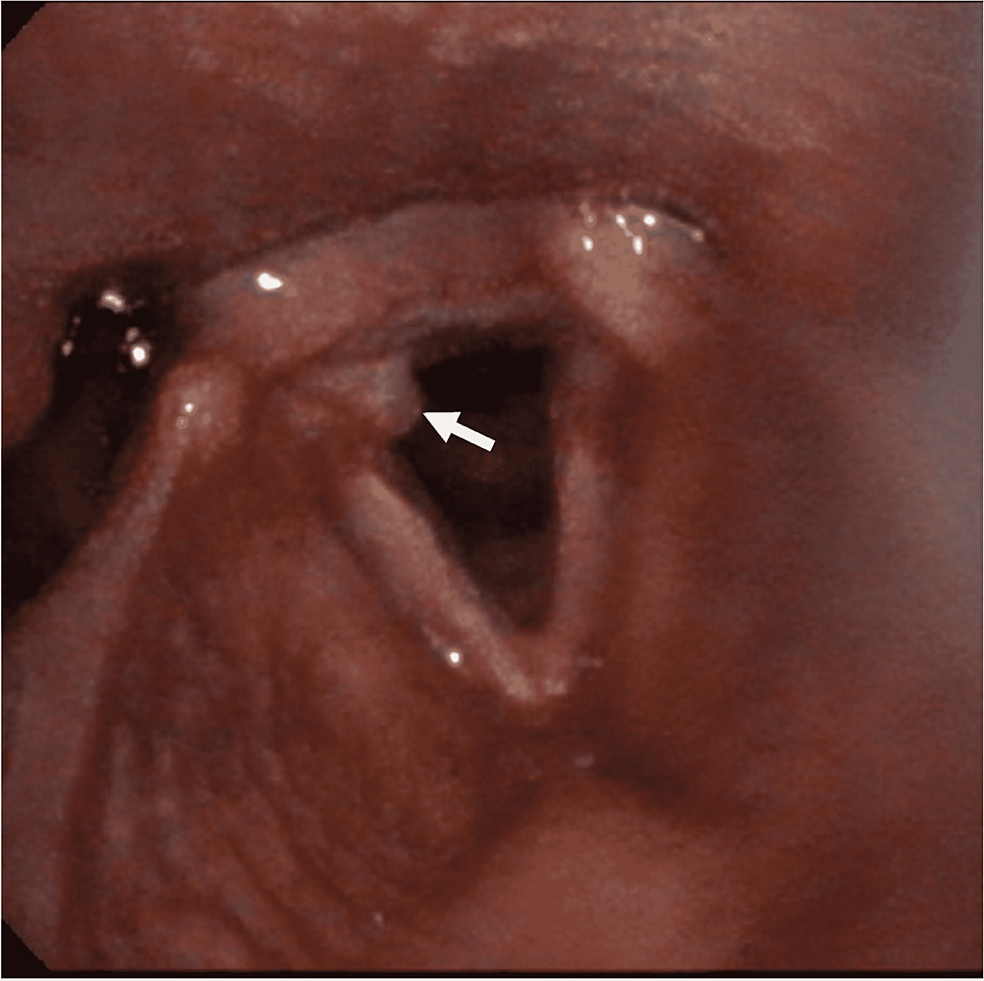
Granuloma of the right vocal fold Flexible laryngoscopy image of vocal fold granuloma on the posterior third of the right vocal fold (white arrow indicates the location of the granuloma).

On physical exam, the patient was in no acute distress and was not actively hiccupping during the exam. He had no lesions in the oral cavity or oropharynx. His neck was soft, and a left thyroid mass was palpated. The rest of the physical exam was unremarkable. On flexible laryngoscopy, there were no lesions detected in the nasal cavity, nasopharynx, oropharynx, supraglottis, or hypopharynx. There was a granuloma found on the posterior third of the right vocal fold (Figure 1). Stroboscopy confirmed a granuloma on the right vocal fold with intact vocal fold mucosal waves and complete glottal closure. The larynx showed significant hyperfunction and squeeze with strong laryngeal breath-holding.

**Figure 2:**
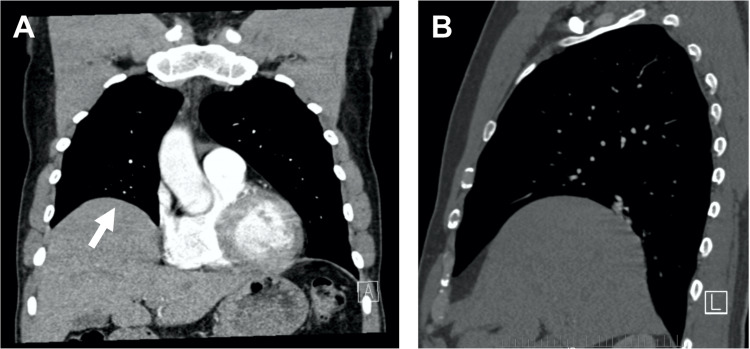
Eventration of the right hemidiaphragm CT scan of the chest demonstrating eventration of the right hemidiaphragm. The white arrow in the coronal view (Panel A) indicates the area of diaphragmatic eventration. Panel B shows a sagittal view of the diaphragmatic eventration.

To further evaluate the etiology of his intractable hiccups, a computed tomography (CT) scan of the chest was requested to further evaluate this finding and to rule out potential neoplastic or infectious causes of his hiccups. CT scan of the chest found eventration of the right hemidiaphragm with an incidentally found enlarged and heterogeneous thyroid with a substernal extension of the left thyroid lobe (Figures 2A, 2B). The patient’s thyroid was evaluated with a dedicated ultrasound, showing a 3 x 3 x 2 cm nodule in the lower pole of the left thyroid gland. This nodule caused no tracheal deviation. Fine needle aspiration (FNA) of the nodule showed no malignant cells. It was determined from this information that the left thyroid lobe nodule was not sufficiently enlarged to cause impingement on the phrenic nerve.

Given these findings, the cause of the patient’s hiccups was determined to be the eventration of his right hemidiaphragm. Potential neoplastic or infectious causes of his hiccups were sufficiently ruled out with the imaging findings. The patient was continued on an increased dose of baclofen for hiccup control. He was referred to voice therapy to work on rescue breathing for laryngospasm and to reduce laryngeal hyperfunction for further management of his hiccups and reduction of his vocal fold granuloma. Finally, the patient was referred to thoracic surgery for further evaluation of his diaphragmatic eventration and to determine whether there is a surgical indication. Thoracic surgery conducted a fluoroscopic observation of his diaphragm during normal respiration and with forced inspiratory “sniff” maneuvers. This showed the decreased movement of the right hemidiaphragm on inspiration and expiration relative to his left hemidiaphragm, but no paradoxical motion during the “sniff” maneuver, thus confirming right-sided diaphragmatic eventration. Thoracic surgery determined that there is no acute indication for surgical intervention at this time and will continue to monitor the patient. The patient was told to follow up with otolaryngology in one month and thoracic surgery as needed, and the patient was amenable to this plan. We anticipate that supportive treatment of his hiccups with the combination of medication, voice therapy, and collaboration with thoracic surgery for potential future surgical indications will lead to improvement of this patient’s hiccups.

## Discussion

Hiccups are merely an inconvenience when transient but can become intolerable when intractable. Hiccups are considered intractable when they persist for over one month [2]. Intractable hiccups have been documented to cause a myriad of sequelae, including sleep deprivation, mood disorders, aspiration pneumonia, and malnutrition [6]. Additionally, when hiccups become intractable, they are often a symptom of potentially serious underlying structural or neoplastic abnormalities causing irritation of the phrenic nerve or direct diaphragmatic irritation [2-4]. It is therefore imperative to perform a thorough workup for intractable hiccups and to better understand the potential causes of this symptom.

Hiccups are caused by an involuntary contraction of inspiratory muscles leading to a sudden closure of the glottis [1]. The pathogenesis of a hiccup is centered around irritation of phrenic or vagus nerve branches or direct irritation of the diaphragm that causes activation of the hiccup reflex arc, which ends when the cause of the irritation resolves. The differential diagnosis for intractable hiccups is large and requires a thorough history, physical examination, and proper imaging for a full evaluation. The possible causes all affect the hiccup reflex arc. For the otolaryngologist, the causes of intractable hiccups most important to keep in mind include neck cysts, pharyngitis, laryngitis, esophagitis, esophageal lesions, and GERD [7]. All the aforementioned causes could potentially irritate the branches of the vagus nerve, particularly the tympanic and pharyngeal branches of the vagus nerve [2]. Herpesvirus laryngitis and esophagitis in particular have been known to cause intractable hiccups and it is important to rule out these etiologies [8,9]. While our patient did not receive viral titers, his upper endoscopy performed by GI and his flexible laryngoscopy were both without abnormalities except for the right vocal fold granuloma. This was deemed sufficient to rule out these infectious causes.

In the thoracic cavity, hiccups can be caused by phrenic nerve irritation or direct diaphragmatic irritation from neoplasms, infectious pneumonia, pericarditis, and even myocardial infarction [1]. In this reported case, we have shown that diaphragmatic eventration should be added to the differential as another thoracic cavity cause of intractable hiccups. To our knowledge, this is the first reported case of intractable hiccups caused by diaphragmatic eventration. After a thorough workup that eliminated other neoplastic or infectious causes of his hiccups, diaphragmatic eventration was determined to be the cause.

Diaphragmatic eventration is an abnormal elevation of the hemidiaphragm caused by the replacement of diaphragm musculature with thin, fibro-elastic tissue without defects in continuity [10]. Eventration can be caused by congenital or acquired causes [11]. Congenital causes of eventration result in abnormal diaphragmatic muscle development from birth and include spondylocostal dysostosis, Kabuki syndrome, Beckwith-Wiedemann syndrome, Poland syndrome, pulmonary hypoplasia, spinal muscular atrophy, and congenital heart disease, among many others [11]. Acquired causes of eventration are more common than congenital causes and are most often idiopathic [12]. Eventration can also be secondary to infectious or neoplastic etiologies that result in phrenic nerve injury and muscle atrophy [11,12]. Since this patient has no significant past medical history suggestive of a known congenital or acquired cause of his eventration, it is likely that his diaphragmatic eventration was acquired idiopathically. Most patients, including this patient, have diaphragmatic eventration discovered incidentally on chest X-ray and the most common presenting symptom is dyspnea on exertion [10,11]. Other symptoms can include orthopnea, tachypnea, recurrent respiratory infections, dyspepsia, dysphagia, and GERD [10,11]. This patient had GERD, which could have also been caused by his eventration, and this was successfully resolved with anti-reflux medication.

Treatment for this patient consisted of a multi-disciplinary approach, given the complex and rare nature of his disease process. Typically, treatment of intractable hiccups consists of treating the underlying cause while supplementing with medications [5,13]. In this case, the underlying cause was diaphragmatic eventration, which is treated with supportive care in mild cases and with surgical plication of the diaphragm in severe cases [11,14]. Supportive care includes oxygen supplementation and pulmonary rehabilitation in patients with hypoxemia if indicated [11]. Surgical plication is indicated in patients in whom medical management has failed, and a study by Celik et al. showed remission of symptoms and quality of life improvement in 13 patients who underwent unilateral diaphragmatic plication [14]. As this patient has not yet failed medical management, we decided to treat the patient with a combination of supportive medical therapy for his hiccups and voice therapy to help reduce his vocal fold granuloma. Baclofen and gabapentin are the most common pharmacological treatments for intractable hiccups and the use of baclofen is supported by small randomized controlled trials [13]. This patient was initially treated with a low dose of baclofen before coming to our service without successful remission of his symptoms, so it was decided to increase the dose and observe for improvement.

Voice therapy is an important component of this patient’s treatment plan that has been previously shown to help patients with intractable hiccups [7]. A study conducted by Martinez Paredes et al. was the first to demonstrate an association between intractable hiccups and vocal fold granuloma, and our patient is another example of this association [7]. Our patient had a single vocal fold granuloma located in the posterior right vocal fold, likely caused by constant phonotrauma secondary to the frequent glottic closures elicited by his hiccups [15]. The vocal lesion itself can contribute to laryngeal hyperfunction seen in this patient via compensatory muscle tension [15]. Martinez Paredes et al.’s patient had bilateral vocal fold granulomas secondary to intractable hiccups and showed significant improvement of hiccups after nine months of voice therapy, which incorporated rescue breathing techniques, jaw release strategies, tongue resting posture techniques, and singing exercises [7]. Our patient also demonstrated significant laryngeal breath-holding and hyperfunction secondary to his intractable hiccups and was determined to potentially benefit from voice therapy.

## Conclusions

Intractable hiccups can be a debilitating symptom for patients and are often due to a more serious underlying condition. The cause of our patient’s intractable hiccups was diaphragmatic eventration, and this resulted in a multi-disciplinary treatment plan including baclofen, voice therapy, and collaboration with thoracic surgery. To our knowledge, this is the first case reported of diaphragmatic eventration causing intractable hiccups. It is important that diaphragmatic eventration is considered in the broad differential for intractable hiccups.
